# *In vitro* assessment of the digestibility of forage based sheep diet, supplemented with raw garlic, garlic oil and monensin

**Published:** 2012

**Authors:** Ehsan Anassori, Bahram Dalir-Naghadeh, Rasoul Pirmohammadi, Akbar Taghizadeh, Siamak Asri-Rezaei, Safa Farahmand-Azar, Maghsoud Besharati, Morteza Tahmoozi

**Affiliations:** 1 *Department of Clinical Sciences, Faculty of Veterinary Medicine, Urmia University, Urmia, Iran;*; 2 *Department of Animal Sciences, Faculty of Agriculture, Urmia University, Urmia, Iran; *; 3 *Department of Animal Sciences, Faculty of Agriculture, University of Tabriz, Tabriz, Iran.*

**Keywords:** Daisy^II ^, Essential oil, Garlic, Gas production, Monensin

## Abstract

The effect of inclusion of garlic essential oil (EO) at 33, 66 and 100 µg mL^-1^, raw garlic (GAR) at 5, 10 and 15 mg mL^-1^ and monensin (MON) at 7.5 µg mL^-1^ of incubation medium on organic matter digestibility (OMD) was studied with *in vitro* gas production, ANKOM daisy^II^ and conventional *in vitro* (IVOMD) methods. The material was incubated with sheep ruminal fluid and the experimental design was a completely randomized design. Cumulative gas production was recorded at 0, 2, 4, 6, 8, 12, 16, 24, 36, 48, 72 and 96 hours of incubation. Conventional *in vitro* OMD was determined after 48 hours incubation in acid and pepsin solutions. Samples for Daisy^II^ OMD prepared according to the operating instructions supplied by ANKOM^®^ Tech. Co., Fairport, USA. Compared to *in vitro* dry matter digestibility (IVDMD), Daisy^II^ and gas production techniques overestimated (*P* < 0.05) OMD. The addition of EO and MON reduced (*P *< 0.05) the organic matter (OM), neutral detergent fiber (NDF), acid-detergent fiber (ADF) digestion, and gas production volume. The potential of gas production and rate of gas production for EO and MON were the lowest; however, these variables were higher for GAR supplemented groups. It was concluded that raw garlic could be of great interest for its usage as a modulator of ruminal fermentation.

## Introduction

Many attempts have been made to modify ruminant digestion to improve animal productivity. Monensin ionophor antibiotic has been used widely in ruminants to modify rumen fermentation. Monensin has selective antimicrobial activity and its supplementation enhances feed efficiency by inhibiting most lactate-producing ruminal bacteria, increasing the ruminal pH in animals consuming high-grain diets and decreasing the acetate/propionate ratio.^1^ These effects are also accompanied by a decrease in methane production.^2^ However, using monensin as a feed additive have raised welfare and public health concerns because it can lead to the development of resistance to antibiotics both in animals and human beings. Therefore, a goal for nutritionists in this area would be to find new additives to modulate microbial activity in the rumen. Among the alternatives, plant extracts are being studied because they are known to contain active compounds with numerous effects including antimicrobial activity.^3^ The essential oil of garlic has been shown to possess a special feature of having unique compounds which do not exist in the original plant and are produced from thiosulfates during the steam treatment.^4^ They are very active against a wide range of gram-positive and gram-negative bacteria, fungi, parasites, and viruses.^5^ The present study was set out in three stages to compare digestibility estimates of forage based ruminant diets using the conventional *in vitro* OM digestibility, Daisy^II^ method and *in vitro* gas production techniques. Measurement of *in vitro* OM digestibility has been widely used to assess the nutritional quality of feeds, due to its high correlation with *in vivo* digestibility. Development of the ANKOM incubator and fiber apparatus (ANKOM Technology Corp., Fairport, NY, USA) have greatly improved labor efficiency by allowing large numbers of feeds to be simultaneously assayed. Both *in vitro* gas production and the ANKOM devices can be used for rapid evaluation of nutritional quality of feeds. 

The aim of this study was to assess the influence of raw garlic bulb (*Allium sativum*) and garlic essential oil on ruminal organic matter digestibility, in comparison with monensin (MON).

## Materials and Methods

**Animals, feed samples and rumen fluid collection. **Rumen fluid was obtained from the rumen of four, 3-year-old wether of similar weight fitted with rumen fistula. The substrate used for the *in vitro* procedures (Table 1), were collected from a ration prepared for wethers, to meet the maintenance energy and protein requirements with an estimated metabolizable energy of approximately 10.5 MJ kg^-1^ dry matter (DM). ^6^ The ration was fed twice per day at 08:00 and 16:00. The wethers had free access to fresh drinking water. A sample of ruminal contents was collected 3 hours (h) post morning meal in thermos flasks and taken immediately to the laboratory where it was strained through various layers of cheesecloth and kept at 39 ˚C under a CO_2_ atmosphere. Feed samples dried in a 60 ˚C forced air oven. Samples were ground to pass a 1mm sieve in a Wiley mill and used for chemical analysis, *in vitro* gas production, Daisy^II^ and *in vitro* OM digestibility. All experimental procedures were approved by the Ethic Committee of Research Council (Urmia University).

**Additives. **Raw garlic bulb harvested from Hamadan, Iran. Garlic oil was prepared according to Clevenger *et al*.^7^ In brief, the cloves of garlic were sliced and crushed and then subjected to hydro distillation for 3 h using a Clevenger type apparatus. The oil was transferred into dark glass bottles, capped and stored in a refrigerator (4 ˚C). Monensin was obtained from a pharmaceutical company.^a^

Eight treatments on total digestion media were carried out: no additive, as a negative control (CTR); the monensin antibiotic, as a positive control, 7.5 µg mL^-1^ (MON); GAR bulb, at three levels; 5mg mL^-1^ (GAR_1_), 10 mg mL^-1^ (GAR_2_) and 15 mg mL^-1^ (GAR_3_) and garlic EO included 33 µg mL^-1^ (EO_1_), 66 µg mL^-1^ (EO_2_) or 100 µg mL^-1^ (EO_3_). All treatments and doses were dissolved in ethanol, and the control was also dosed with the equivalent amount of ethanol (0.015 mL) and then diluted with mineral buffer. The doses of monensin were calculated based on other studies for antimicrobial activity to meet or exceed the minimum inhibitory concentrations (MICs).^8^^,^^9^ The doses of raw garlic and garlic EO were according to the previous studies.^10^^-^^12^

**Chemical composition. **Feed samples dry matter (DM, method ID 934.01), ash (method ID 942.05), crude protein (CP, method ID 984.13), ether extract (EE, method ID 920.30) and organic matter (OM, method ID 942.05) were determined by procedures of AOAC.^13^ The NDF and ADF concentrations were determined using the methods of Van Soest *et al*.^14^ with the addition of sodium sulfite. The NDF and ADF procedures were adapted for use in an ANKOM 200 Fiber Analyzer. Residue was ashed to allow NDF and ADF to be presented as a proportion of OM.The GC–MS analyses of the garlic oil were carried out using a Finigan gas chromatography^b^ coupled with mass spectrometry.^c^ Data were processed by Xcalibur.^b^ Retention index for each 

peak/compounds was calculated by comparing its retention time against those of alkane series (C8-C40, corresponding retention index values from 800 to 4000). All compounds were identified by comparing both the MS spectra and retention index with those available in libraries, i.e. NIST, Wiley, and internally compiled spectra libraries. 

**Statistical analysis.** Data were transferred to a Microsoft Excel spreadsheet (Microsoft Corp., Redmond, WA, USA). Using SPSS 16.0 statistical software (SPSS Inc., Chicago, IL, USA), a Pearson chi-square test and Fisher's exact two-tailed test analysis was performed and differences were considered significant at values of *P* < 0.05.

**Tilley and Terry two-stage** ***in vitro*** **(conventional** ***in vitro*** **or IVOMD).** *In vitro* OM digestibility was estimated using the acid-pepsin digestion method based on Tilley and Terry.^15^ The buffer solution was the McDougall ‘synthetic saliva’, with pH 6.9.^16^ Ruminal inoculum obtained from sheep and incubating triplicate 0.5 g samples supplemented with additives followed by 48 h acid-pepsin incubation.^15^

***In vitro*** **digestion with Daisy** ^II^ **method. **The Daisy^II^ incubation method involves digestion of ground plant biomass in buffered rumen fluid for 48 h (in a Daisy^II^ Incubator), followed by NDF digestion in an ANKOM 200/220 Fiber Analyzer. The method was conducted according to the operating instructions supplied by ANKOM. Dacron bags with a pore size of 50 µm were used for incubation of samples for OM digestibility. Bags were rinsed with acetone and dried in a forced air oven at 60 ˚C for 6 h. Ground samples (0.25 g per bag) were weighed into triplicate bags, heat sealed and placed in digestion jars. The buffered, rumen fluid solution preparation was based on that of Tilley and Terry.^15^ At the end of the 48 h incubation, jars were removed from the chamber, the incubation solution was discarded, and bags rinsed four times with distilled water.

For *in vitro* true digestibility determination, bags were placed in an ANKOM fiber analyzer and boiled in neutral detergent solution for 75 min. Bags were removed, soaked twice in acetone for 5 min at each soaking and dried at 100 ˚C for 24 h. The *in vitro* true digestibility (IVTD) was calculated as the difference between OM incubated and the residue after neutral detergent (ND) treatment. *In vitro* fiber digestibility was calculated as the difference between the amount of fiber incubated and recovered after ND treatment.

***In vitro*** **gas production. **Ruminal fluid was collected approximately 3 h after morning feeding from two fistulated sheep consuming maintenance level of energy and protein (Table 1). Ruminal fluid was immediately squeezed through four layers of cheesecloth and was transported to the laboratory in a sealed thermos. The resulting ruminal fluid was purged with deoxygenated CO_2_ before use as the inoculum. Gas production was measured by Fedorak and Hrudy method.^17^ Approximately 300 mg of dried and ground (2 mm) samples was weighed and placed into serum bottles. Buffered rumen fluid with McDougall’s buffer (20 mL) was pipetted into each serum bottle.^16^ The gas production was recorded after 2, 4, 6, 8, 12, 16, 24, 36, 48, 72, and 96 h of incubation. Total gas values were corrected for the blank incubation, and reported gas values are expressed in mL per 1 gram of DM. Rate and extent of gas production was determined for each feed by fitting gas production data to the non-linear equation *Y *= *a*+*b (*1 − e^−^^ct^*)*,^18^ where *y *is the volume of gas produced at time *t*, *a*+*b *the gas production of soluble and insoluble fraction (mL g^−1^ DM), and *c *the fractional rate of gas production. Variables *b *and *c *were estimated by an iterative least square method using a non-linear regression procedure of the statistical analysis systems.^19^ Organic matter digestibilities (OMD) were calculated using equations of Menke *et al.*,^20^ as: 

*OMD*_ (__g 100 g_^-1^_ DM)_ *= 14.880 + 0.889×GP + 0.450×CP + 0.0651× XA*

in which, XA is ash in g 100 g^-1^ DM and GP is the net gas production (mL) at 24 h.

**Statistical analysis. **Data obtained from *in vitro* Tilley and terry, Daisy^II^ and gas production studies was subjected to analysis of variance as a completely randomized design by the GLM procedure of SAS Institute Inc.^19^ and treatment means were compared by the Duncan test. Significance was declared at *P *< 0.05.

## Results

**Raw garlic and garlic oil composition. **The ingredients of substrate have been presented in Table 1. Crude fat of garlic bulb was 7.1 g kg^−1^ on a DM basis. Garlic bulb yielded 0.12% garlic oil on a wet weight basis. The GC–MS analyses of garlic oil revealed that trisulfide di-2-propenyl (32.76%), diallyl disulfide (28.41%) and trisulfide methyl 2-propenyl (14.26%) are the main components of the oil. 

**Tilley and Terry two-stage** ***in vitro*** **method. **Chemical content and OM digestibility estimates for diets supplemented with additives are presented in Tables 1 and 2, respectively. Diets supplemented with MON and EO significantly reduced OM digestibility compared to CTR (*P *< 0.05). Garlic supplementation had no effects on digestibility (*P* > 0.05). Digestibility of substrate supplemented with EO_2_ and EO_3_ was reduced significantly compared to EO_1_ and MON. Organic matter digestibility was greater in gas production technique and Daisy^II^ than IVOMD (*P* < 0.001) in all treatment groups (Table 2).

**Daisy** ^II^ ***in vitro* digestion. **There were differences (*P < *0*.*0001) among feeds in *in vitro* OM and NDF digestibility (Table 2). Organic matter digestibility in Daisy^II^ *in vitro* digestion was similar to conventional *in vitro* acid-pepsin OM digestion (Table 2). OMD was significantly lower for substrates supplemented with MON and EO compared to CTR. The OMD values were highest (*P *< 0.01) for GAR_3_ and CTR and lowest for EO_3_ treatments.

No statistical difference (*P *> 0.05) was detected with the

Daisy^II^ technique for NDF and ADF digestibility of diets supplemented with raw garlic. For diets supplemented with MON and EO, NDF and ADF digestibility decreased significantly (*P *< 0.05) compared to CTR. In addition, a reduction (*P *< 0.05) in NDF and ADF degradability by EO was found especially for diets incubated by high level of essential oils (Table 2). Compared to gas production technique the Daisy^II^ technique overestimated (*P *< 0.05) OMD in EO supplemented groups but no significant effects observed with other treatments.

**Table 1:** Chemical composition of incubated ingredients

**Item**	**Amount**
**Ingredient, % of DM**	
Alfalfa hay	38.14
Corn silage	39.69
Soy bean meal	8.50
Barley grain	10.99
Wheat bran	2.68
**Chemical composition**	
DM, %	71.95
Ash, %	9.18
CP, % of DM	16.50
NDF, % of DM	39.73
ADF, % of DM	20.93

DM = dry matter, CP = crude protein, NDF = neutral detergent fiber, ADF = acid detergent fiber

**Table 2:** Comparison of organic matter digestibility (OMD, %) for diet supplemented with monensin, raw garlic and garlic EO with conventional *in vitro* (Tilley and Terry), Daisy^II^ and gas production techniques

**Techniques**	**Treatments**										
	CTR		MON	GAR_1_	GAR_2_	GAR_3_	EO_1_	EO_2_	EO_3_	SE	*P* value
**Tilley and Terry method**											
OMD	46.87^a^,^y^		38.32^b^,^y^	45.41^a^,^y^	45.47^a^,^y^	46.20^a^,^y^	39.47^b^,^y^	35.01^c^,^y^	34.49^c^,^y^	0.79	<0.0001
**Gas production method**											
OMD	57.38^a^,^x^		51.21^b^,^x^	55.48^a^,^x^	56.56^a^,^x^	56.44^a^,^x^	39.40^c^,^y^	32.39^d^,^z^	30.61^d^,^y^	1.28	<0.0001
**ANKOM, DaisyII method**											
OMD		55.13^a^,^x^	50.93^b^,^x^	54.54^a^,^x^	53.85^a^,^x^	55.15^a^,^x^	50.10^b^,^x^	44.48^c^,^x^	44.44^c^,^x^	0.79	<0.0001
NDFD		61.89^a^	57.49^b^	61.70^a^	61.87^a^	62.18^a^	48.78^c^	46.03^d^	41.31^e^	0.64	<0.0001
	
ADFD		54.15^a^	46.16^b^	53.97^a^	54.16^a^	54.88^a^	40.54^c^	31.32^d^	30.08^e^	0.58	<0.0001

CTR = control, MON = monensin 7.5 µg mL^-1^, GAR_1_ = raw garlic bulb 5 mg mL^-1^, GAR_2_ = raw garlic bulb 10 mg mL^-1^, GAR_3_ = raw garlic bulb 15 mg mL^-1^ EO_1_ = garlic essential oil 33 µg mL^-1^, EO_2_ = garlic essential oil 66µg mL^-1^, EO_3_ = garlic essential oil 100 µg mL^-1^

OMD = organic matter digestibility, NDFD = neutral detergent fiber digestibility, ADFD = acid detergent fiber digestibility

a,b,c,d,e The means within a same row without common letter(s) differ (*P* < 0.05).

x,y,z The means within a same column without common letter(s) differ (*P* < 0.05) for organic matter digestibility

**Gas production technique. **There was a similar pattern with two previous methods in OM digestibility of samples; however, EO_1_ decreased significantly OMD compared to MON (*P *< 0.05). The volumes of gas production of the diet supplemented by different additives are shown in Table 3. There were significantly differences in gas production volumes among treatments at different incubation times (*P *< 0.05). Both MON and EO reduced (*P <* 0.05) gas production volume compared to CTR throughout the incubation time (Table 3). After 2 h incubation GAR_3_ had lowest gas production volume among treatments (*P* < 0.001), also in comparison with CTR, MON, EO_1_ and EO_3_ significantly decreased gas production volume (*P *< 0.01). Substrates supplemented with GAR_3_ (*P *< 0.05) caused lower gas production volume than CTR, GAR_1_ and GAR_2_ treatments but only for 6 h of incubation. No significant effect (*P *> 0.05) was observed for GAR_3_ after 8 h of incubation. GAR_1_ and GAR_2_ didn’t differ in patterns of gas production, although GAR_1_ differed from CTR starting from about 48 h of incubation (Table3). The amounts of potential gas production (a+b) for CTR and GAR_2_ treatments were higher than other treatments (Table 3). The EO_3_ treatment among treatments had the lowest (a+b) fraction (*P *< 0.05). The rate constant of gas production (c) for GAR_1_ and GAR_3_ was highest among the treatments (0.043 and 0.04 mL per hour, respectively). The pattern of fermentation of garlic EO was distinctly different from MON and GAR, particularly in the end of incubation (Table 3).

**Table 3:** Gas production of feed stuffs incubated in buffered rumen fluid with different additives (mL g^-1^ DM).

**Treatments**	**Incubation times (h)**												
	2	4	6	8	12	16	24	36	48	72	96	a+b	c (h^-1^)
**CTR**	24.83^a^	32.84^a^	69.10^a^	95.61^a^	119.54^a^	142.60^a^	193.93^a^	224.64^a^	271.60^a^	287.88^a^	309.88^a^	309.08^a^	0.031^cd^
**MON**	19.93^c^	24.14^b^	57.80^b^	83.98^b^	106.93^b^	128.98^b^	159.22^b^	192.19^b^	215.10^ab^	226.27^c^	249.45^c^	243.50^d^	0.032^c^
**GAR**_1_	23.44^ab^	29.26^a^	63.78^ab^	87.23^b^	115.30^ab^	136.56^ab^	183.22^a^	208.82^ab^	224.21^b^	257.62^b^	279.17^b^	272.30^c^	0.04^a^
**GAR**_2_	24.42^a^	32.59^a^	70.04^a^	100.44^a^	122.50^a^	143.54^a^	189.31^a^	223.13^a^	249.00^ab^	286.37^a^	310.59^a^	309.18^a^	0.03^b^
**GAR**_3_	16.00^d^	25.26^b^	61.28^b^	95.75^a^	124.04^a^	145.30^a^	188.63^a^	221.12^a^	243.80^ab^	280.58^a^	299.24^ab^	300.00^b^	0.04^a^
**EO**_1_	20.44^bc^	21.37^b^	21.73^c^	24.64^c^	30.67^c^	61.69^c^	92.80^c^	108.22^c^	123.50^ab^	135.61^d^	147.39^d^	156.61^e^	0.03^c^
**EO**_2_	20.44^bc^	22.12^b^	23.24^c^	27.22^c^	29.59^c^	30.79^d^	53.38^d^	85.28^d^	100.80^b^	107.26^e^	113.48^e^	145.85f	0.01^e^
**EO**_3_	21.78abc	23.60^b^	24.51^c^	25.54^c^	26.98^c^	27.80^d^	43.36^d^	65.97^e^	86.11^b^	104.44^e^	113.00^e^	132.72^g^	0.02^e^
**SEM**	1.82	2.25	3.62	4.13	4.89	5.33	7.23	10.68	10.84	11.61	12.21	0.277	0.00

DM = dry matter, CTR = control, MON = monensin 7.5 µg mL^-1^, GAR_1_ = raw garlic bulb 5 mg mL^-1^, GAR_2_ = raw garlic bulb 10 mg mL^-1^, GAR_3_ = raw garlic bulb 15 mg mL^-1^, EO_1_ = garlic essential oil 33 µg mL^-1^, EO_2_ = garlic essential oil 66µg mL^-1^, EO_3_ = garlic essential oil 100 µg mL^-1^, a+b = potential of gas production mL g^-1^ DM, c = rate constant of gas production during incubation mL h^-1^

a,b,c,d,e,f,g The means within a same column without common letter(s) differ significantly (*P* < 0.05).

## Discussion

The technique of Tilley and Terry, was identified as an essential tool for the evaluation of ruminant feeds and is used widely because of its convenience, particularly when large-scale testing of feed stuffs is required.^15^ The gas measuring technique was considered to be a routine method of feed evaluation after the work of Menke *et al*. where a high correlation between *in vitro* and *in vivo* apparent digestibility was reported for gas production.^20^ Although *in vitro* dry matter digestibility (IVDMD) estimates are different than *in vivo* estimates of DMD, they are, generally, in closer agreement than the newer techniques.

In our study, the Daisy^II^ techniques were used to estimate true digestibility and the conventional *in vitro* and gas production technique were used to estimate apparent digestibility. Theoretically, IVDMD should be expected to have lower values. Also overestimating digestibility for finely ground (1 mm) substrates in the filter bag based technique may be caused by agitation during incubation, boiling in neutral detergent solution, and through rinsing of the filter bags with water after 48 h of incubation. During this procedure a proportion of non-digestible fine particles may have been removed, reducing the weight of residue and increasing the estimate of digestibility compared to the conventional *in vitro *technique in which microbial matter and fine particles are retained. This finding is in accordance with Vogel *et al*.^21^ and Mabjeesh *et al*.^22^

In the present study, raw garlic was shown to have the potential to modify *in vitro* ruminal fermentation and to increase gas production volume compared to monensin. This is in contrast with Cullen *et al*.^23^ who reported that the inclusion of garlic at the higher level caused a significant reduction in dry matter and organic matter digestibilities. It could be explained with garlic’s high oligofructose and inulin content.^24^ Also effects of increased dietary non-starch polysaccharide (NSP) levels on the digestibility of starch, proteins and fat have been demonstrated in pig.^25^ This discrepancy could be explained by stimulating effects of dietary non-starch polysaccharide (NSP) levels on microbial growth in sheep rumen inoculum. In the present study the antimicrobial action of allicin and allin^26^ were not probably enough to be able to inhibit microbial fermentation, although the beneficial bacteria were believed to be unaffected as they were less sensitive to the inhibitory effects of garlic.^24^ The decreased fermentation as indicated by gas production volume for the first 6 h but not at later hours of incubation for GAR_3_, suggests that microbial populations are able to adapt to GAR over time due to shifts in microbial populations or adaptation of individual microbial species to garlic constituents. There are some reports for an adaptive response of rumen bacterial population to essential oil supplementation.^27^ Similarly, Busquet *et al*.^10^ studied effects of garlic oil on *in vitro *rumen microbial fermentation in a 24 h batch culture, although results of present study showed that the beneftial effects of EO did not change over entire experiments. In agreement with our results, Chen *et al*. found higher dry matter and organic matter digestibilities in pigs supplemented with garlic powder^28^; however, the exact mechanisms of this effect remain unclear. 

All additives with the exception GAR decreased NDF degradability in Daisy^II^ digestion method; moreover, in agreement with Wang *et al*., ^9^MON altered *in vitro* ruminal fermentation by reducing OM digestion and lowering gas production. Ionophores, such as monensin, selectively inhibit gram-positive microorganisms,^29^ which include most of the cellulolytic bacteria, capable of hydrolysing fiber (e.g. *Ruminococcus albus,* *Ruminococcus* *flavefaciens,* and *Butyrivibrio fibrisolvens*). In fact, in an *in vitro* degradability experiment, it has been demonstrated that monensin diminished NDF degradation.^30^ In contrast with Busquet *et al*.^31^ and Yang *et al*.^11^ who showed the supplementation of garlic oil did not affect true DM, OM, NDF, and ADF digestibilities, our results showed suppressing effects of EO on OM, NDF and ADF digestibility. These effects could be related to the antibacterial properties of essential oils that decreased the activity of the ruminal microorganism. However, because of the large number of different groups of chemical compounds present in EO, the antibacterial activity cannot be easily attributed to a specific mechanism. Several mechanisms have been suggested to explain the antibacterial action, including degradation of the cell wall, damage to the cytoplasmatic membrane, leakage of cell contents, coagulation of cytoplasm, and depletion of the proton motive force.^32^

NDF degradability was decreased by EO and monensin in the present study. Jalc *et al*. also showed that monensin decreased the *in vitro* degradability of NDF.^30^ Ionophores, such as monensin, act by interrupting trans-membrane movement and the intracellular equilibrium of ions, exhausting and causing the death of some micro-organisms.^33^ In *in vivo* studies, monensin did not affect NDF degradation^1^, because the expression of such effect in an *in vivo* ruminal medium is possibly more complex, inhibiting bacteria such as *Streptococcus bo*v*is *(lactic acid producers) and therefore preventing the pH from falling, favoring activity of the cellulolytic bacteria. Therefore, it could be hypothesized that the effects of essential oils on degradability could be different between *in vitro* and *in vivo* studies. In contrast with Kongmun *et al.*^34^ who reported that garlic powder at 80 g kg^-1^ DM, increased *in vitro* potential degradability of OM in gas production technique, no change observed for OM digestion with gas production and ANKOM daisy^II^ procedure in our study. 

In this study the potential rate of gas production and rate of gas production was the lowest with EO supplementation. Antimicrobial activities of EO have been demonstrated against a wide variety of microorganisms, including Gram-positive and Gram-negative bacteria. The antimicrobial activity of EO has been attributed to a number of terpenoid and phenolic compounds,^35^ as well as the chemical constituents and functional groups contained in the EO, the proportions in which they are present and the interactions between them.^36^ Essential oils have a high afﬁnity for lipids of bacterial cell membranes due to their hydrophobic nature, and their antibacterial properties are evidently associated with their lipophilic character. Nagy and Tengerdy^37^ observed that EO extracted from Sagebrush (*Artemesia tridentata*) markedly inhibited activity of ruminal bacteria *in vitro*. 

Evans and Martin observed that thymol (400 mg L^-1^), a main component of EO derived from Thymus and Origanum plants, was a strong inhibitor of methane *in vitro*, but it also caused a decrease in acetate and propionate concentrations.^38^ Busquet *et al.* found that in batch culture garlic oil and diallyl disulﬁde (300 mg L^-1^ of ruminal ﬂuid) reduced methane production by 74 and 69% respectively, without altering digestibility.^10^

Our results showed that in comparison with monensin, GAR supplementation increased total gas production; however, when compared to CTR there was a decrease in gas production. Wanapat *et al*. found that the supplementation of diet with garlic powder resulted in a reduction of total volatile fatty acids (VFAs) and the proportion of acetate, but the proportion of propionate and butyrate were increased.^12^ Some studies have reported that EO addition increased VFA production,^39^^,^^40^ whereas others showed no effect,^3^^,^^41^ reflecting dependence of the outcome on the dose and diet.^42^ Gas production is an indication of quantitative VFA production. Since truly digested substrate is partitioned among VFA, gas, and microbial biomass; gas measurements only account for substrate that is used for VFA and gas production and does not reflect substrate utilized for microbial growth. 

In conclusion, our results showed that raw garlic like monensin resulted in no adverse effects on *in vitro* digestibility of the diets. However, garlic EO, irrespective of its level, decreased gas production, OM and NDF degradability. The effects of essential oils on fermentation would not be nutritionally beneficial to the rumen energetic metabolism. However, further studies are required to investigate the effects of garlic constituents on *in vivo* ruminal fermentation and animal productivity.

a. Behroodatrak Company, Iran, Rumensin premix licensed by Elanco Division, Eli Lilly Canada Inc

b. Thermo Finigan, USA

c. TRACE 2000 / EI quadrapole
